# Automated Measurement of Speech Recognition, Reaction Time, and Speech Rate and Their Relation to Self-Reported Listening Effort for Normal-Hearing and Hearing-Impaired Listeners Using various Maskers

**DOI:** 10.1177/23312165241276435

**Published:** 2024-09-23

**Authors:** Inga Holube, Stefan Taesler, Saskia Ibelings, Martin Hansen, Jasper Ooster

**Affiliations:** 1 Institute of Hearing Technology and Audiology, 597470 Jade University of Applied Sciences, Oldenburg, Germany; 2 Cluster of Excellence Hearing4all, Oldenburg, Germany; 3 Communication Acoustics, 11233 Carl von Ossietzky University, Oldenburg, Germany

**Keywords:** speech intelligibility, verbal response time, listening effort, hearing impairment, fluctuating maskers

## Abstract

In speech audiometry, the speech-recognition threshold (SRT) is usually established by adjusting the signal-to-noise ratio (SNR) until 50% of the words or sentences are repeated correctly. However, these conditions are rarely encountered in everyday situations. Therefore, for a group of 15 young participants with normal hearing and a group of 12 older participants with hearing impairment, speech-recognition scores were determined at SRT and at four higher SNRs using several stationary and fluctuating maskers. Participants’ verbal responses were recorded, and participants were asked to self-report their listening effort on a categorical scale (self-reported listening effort, SR-LE). The responses were analyzed using an Automatic Speech Recognizer (ASR) and compared to the results of a human examiner. An intraclass correlation coefficient of *r *= .993 for the agreement between their corresponding speech-recognition scores was observed. As expected, speech-recognition scores increased with increasing SNR and decreased with increasing SR-LE. However, differences between speech-recognition scores for fluctuating and stationary maskers were observed as a function of SNR, but not as a function of SR-LE. The verbal response time (VRT) and the response speech rate (RSR) of the listeners’ responses were measured using an ASR. The participants with hearing impairment showed significantly lower RSRs and higher VRTs compared to the participants with normal hearing. These differences may be attributed to differences in age, hearing, or both. With increasing SR-LE, VRT increased and RSR decreased. The results show the possibility of deriving a behavioral measure, VRT, measured directly from participants’ verbal responses during speech audiometry, as a proxy for SR-LE.

## Introduction

In hearing health care, hearing disabilities are often examined using speech-recognition tests. Typically, words or sentences are presented in quiet or in stationary maskers, and the signal level or the signal-to-noise ratio (SNR) for a speech-recognition score of 50% (Speech Recognition Threshold, SRT) is determined. However, stationary maskers and listening conditions with a speech-recognition score of 50% are not often observed in everyday life (e.g., Smeds et al., 2015; Wu et al., 2018). To increase the ecological validity of speech tests (Keidser et al., 2020), stationary maskers can be replaced by fluctuating maskers (e.g., competing speech, modulated maskers) and the SNR can be increased above SRT. At higher SNR, speech-recognition scores can reach a saturation of 100%, while listeners may still experience some degree of listening effort if the task is not too easy (e.g., Krueger et al., 2017b; Slade et al., 2021). Since listening effort cannot be equated with speech-recognition scores (Winn & Teece, 2021), and shows a certain variance while the scores are at ceiling, the measurement of listening effort involved in speech perception at SNRs above SRT is therefore relevant. The question arises as to how listening effort can be measured simultaneously with speech recognition during a speech test, and whether this could be supported using an automated system. Hence, the current contribution compares self-reported listening effort (SR-LE), speech-recognition scores, and the timing of listener responses as a behavioral measure in a speech test. Speech-recognition scores and timing were measured by a human examiner and an Automatic Speech Recognizer (ASR). The experiment was conducted using stationary and fluctuating maskers at several SNRs at and above SRT for young listeners with normal hearing, and older listeners with hearing impairment.

For listeners with normal hearing, speech recognition in fluctuating maskers results in lower SRTs than in stationary maskers (Holube, 2011; Rhebergen et al., 2006). This advantage is attributed to dip-listening in fluctuating maskers (Moore, 2008), an ability that is reduced or absent for listeners with hearing impairment (e.g., Duquesnoy, 1983; Festen & Plomp, 1990; Gustafsson & Arlinger, 1994; Irsik et al., 2022; Wagener & Brand, 2005). George et al. (2006) ascribed the difficulties of hearing-impaired listeners in fluctuating maskers mainly to audibility, temporal resolution, and age. In addition to lower SRTs, shallower slopes of the psychometric function were observed in fluctuating compared to stationary maskers (see e.g., MacPherson & Akeroyd, 2014; Wagener & Brand, 2005). The review of MacPherson and Akeroyd (2014) only tentatively concluded that there were effects of age and hearing impairment on the slope.

The effect of masker type, age, and hearing impairment on listening effort is less clear. Listening effort was defined by McGarrigle et al. (2014) as “the mental exertion required to attend to, and understand, an auditory message” and by Pichora-Fuller et al. (2016) as “the deliberate allocation of mental resources to overcome obstacles in goal pursuit when carrying out a listening task.” When applied to our research questions, the listening task was a speech-recognition test.

Several methods to measure listening effort have been proposed (see the reviews of Francis & Love, 2020; Gagné et al., 2017; Klink et al., 2012a, 2012b; McGarrigle et al., 2014; Pichora-Fuller et al., 2016; Richter et al., 2023). The methods can be divided into physiological measures, behavioral measures, and self-reports. Physiological measures include pupil dilation (e.g., Koelewijn et al., 2012; Zekveld et al., 2010), skin conductance (e.g., Holube et al., 2017; Mackersie & Cones, 2011), heart-rate variability (e.g., Seeman & Sims, 2015), electroencephalography (EEG), and functional magnetic resonance imaging (fMRI). Behavioral measures are obtained in single-task or dual-task paradigms. In single tasks, reaction times are measured when listeners press a response button (e.g., Hecker et al., 1966; Houben et al., 2013; Mackersie et al., 1999; Pratt, 1981; Prodi & Visentin, 2019; Prodi & Visentin, 2022; Tun et al., 2010; van den Tillaart-Haverkate et al., 2017; Visentin & Prodi, 2018) or give a verbal response (e.g., Davis et al., 1961; Gustafson et al., 2023). In dual-task paradigms, the (decreased) performance in a secondary task is measured while doing a primary task, which is typically a speech test (see e.g., Downs, 1982; Hicks & Tharpe, 2002, and the reviews by Anderson Gosselin & Gagné, 2010 and Gagné et al., 2017). Listening effort can also be obtained with self-reports using questionnaires (e.g., questions from the Speech, Spatial, and Qualities of hearing scale, SSQ; Gatehouse & Noble, 2004) or subjective assessments on rating scales (e.g., Feuerstein, 1992; Luts et al., 2010). The current contribution focuses on self-reports on a rating scale and verbal responses in a single-task experiment.

Initially, approaches for self-reports on rating scales asked listeners to rate their perceived ease of listening on a scale from 0 to 100 (Feuerstein, 1992; Humes et al., 1999). Morimoto et al. (2004) proposed using listening difficulty, instead of the ease of listening. They used a scale with four categories to measure listening difficulty in public spaces at the higher SNRs that are experienced in everyday life. A more detailed categorical scale for listening effort with seven categories ranging from “no effort” to “extreme effort” and supplemented by intermediate steps, was used by Luts et al. (2010) and Krueger et al. (2017a). In line with the assessment of ease of listening, listening effort has also been rated on a scale from 0 to 100 (e.g., Desjardins & Doherty, 2013; Giuliani et al., 2021), or on a scale from 0 to 10 including labeled categories (e.g., Hällgren et al., 2005; Larsby et al., 2005; Zekveld et al., 2010, 2011), or using a visual-analog scale with labeled endpoints (e.g., Mackersie & Cones, 2011; Rudner et al., 2012; Seeman & Sims, 2015; Strand et al., 2018).

In addition to the differences in the instructions for self-reports (ease of listening, listening difficulties, listening effort), the outcomes of self-reports may be influenced by the interpretation of the task by the listeners. Feuerstein (1992) already speculated whether the perceived ease of listening was influenced by task performance. Moore and Picou (2018) showed that listeners may substitute a complex question, in their publication the effort it took to complete the task, by an easier question, e.g., their perceived performance. However, Giuliani et al. (2024) concluded that task performance alone was not able to explain listening effort ratings. Another explanation assumes that listening difficulty may be assessed instead of listening effort (Rovetti, 2022).

An intuitively clear finding was reported for SR-LE, SNR, and speech-recognition scores: SR-LE decreases with increasing SNR (e.g., Giuliani et al., 2021; Krueger et al., 2017b) and with increasing speech-recognition scores (e.g., Krueger et al., 2017b; Mackersie & Cones, 2011; Zekveld et al., 2010, 2011). For masker type, hearing impairment, and age, the findings are less clear: Several studies reported higher SR-LE in fluctuating maskers than in stationary maskers (Desjardins & Doherty, 2013; Hällgren et al., 2005; Larsby et al., 2005; Rudner et al., 2012). However, Krueger et al. (2017b) showed lower SR-LE and higher speech-recognition scores for fluctuating, rather than stationary, maskers at the same SNR. When fitting a curve on SR-LE relative to SNR, they also observed shallower slopes for fluctuating maskers compared to stationary maskers. In several studies, higher SR-LE was reported for hearing-impaired listeners than for normal-hearing listeners (e.g., Krueger et al., 2017b; Larsby et al., 2005; Zekveld et al., 2011). Desjardins and Doherty (2013), however, observed lower SR-LE (higher ease of listening) for hearing-impaired older listeners relative to normal-hearing older listeners. Hällgren et al. (2005) and Larsby et al. (2005) did not show an effect of age on SR-LE, while at the same speech-recognition score Zekveld et al. (2011) observed lower SR-LE for older than for younger listeners, both with normal hearing. These diverse findings preclude reaching predictions as to whether a difference in SR-LE can be expected between young normal-hearing listeners and older hearing-impaired listeners and between stationary and fluctuating maskers.

The current contribution focused on the simultaneous analysis of the verbal responses in a single-task experiment and investigated whether this allows to quantify listening effort. Cowan (1992) and Cowan et al. (2003) analyzed verbal responses of children in short-term memory and working memory tasks to measure the underlying cognitive processes. They differentiated between the total duration of the response, i.e., from the end of the stimulus to the end of the response, and its constituent parts, i.e., the preparation time of the response, the word durations, and the inter-word pauses during the response. The timing of the responses was determined by rehearsal for keeping items in the phonological buffer, response planning, memory search and retrieval, and redintegration. Cowan (1992) and Cowan et al. (2003) demonstrated relations between timing parameters and individual performance in the span tasks for children. The findings were replicated for adults by Mattys et al. (2018), who showed that the speed of responses for each item in digit-span and word-span tasks correlated with the span performance, i.e., the number of correctly repeated items. Cowan (1992) concluded that the total duration of the response may be the most sensitive measure of processing speed. When analyzing verbal responses in auditory tasks, however, the response time for the first item (preparation time) is mostly used (e.g., Gatehouse & Gordon, 1990). Hence, the total response duration in the current contribution was divided into the preparation time, called Verbal Response Time (VRT) in the following, and the length of the utterance, characterized by the response speech rate (RSR), i.e., the number of words articulated per second, including pauses. This separation might allow estimation of whether the utterance in terms of RSR provides information beyond the VRT. Pals et al. (2015) concluded that VRT “seems to be a good candidate for a measure of listening effort, complementing speech tests, in research and clinical settings,” and has been shown to have a sufficient test–retest reliability (Gustafson et al., 2023).

Donders (1969) reviewed experiments from as early as 1868 to estimate the speed of mental processes using a so-called “phonautograph.” Different vowels were articulated by a speaker, and the VRT of a listener measured. When the articulated vowel was known, VRT was on the order of 200 ms. For unknown vowels, the VRT increased by about 80 ms, which quantifies the decision process. A similar VRT of about 200 ms was observed by Fry (1975) for vowels, and by Davis et al. (1961) for numbers, letters, and nonsense syllables. Davis et al. (1961) concluded that the number of response alternatives in the repetition task had only a small influence on VRT. Pollack and Rubinstein (1963), however, found that when increasing the number of response alternatives for monosyllabic words, VRT increased up to approximately 700 ms. They also observed a relation of VRT to word-recognition score. Gatehouse and Gordon (1990) used VRT to words and sentences to show the benefit of amplification. They observed an increase in VRT with a decrease in speech-recognition score, and shorter VRT with hearing aids than without hearing aids. Significant changes in VRT were demonstrated for speech-recognition scores close to 100%, where improvements in speech-recognition scores due to hearing aids are difficult to detect. Broadbent (1958) noted that discrimination between stimuli is easier, and therefore faster, when the stimuli are more distinguishable. Hence, higher mental effort and longer VRT are required for degraded stimuli, even if the message was understood. The increase in VRT with the difficulty of the listening condition is in line with both the Ease of Language Understanding (ELU) model (Rönnberg et al., 2013), and the Framework for Understanding Effortful Listening (FUEL; Pichora-Fuller et al., 2016), at least for those listening conditions in which the listeners have not yet given up.

In the past ten years, it was frequently shown that VRT decreases with both increasing SNR and increasing speech-recognition score (Cristiani & Alonso, 2022; Gustafson et al., 2023; Ibelings et al., 2022; Lee & Lee, 2023; Lewis et al., 2016; McGarrigle et al., 2019; Meister et al., 2018; Pals et al., 2015; Visentin et al., 2022). VRT also depends on the type of speech material, i.e., words or sentences (Lewis et al., 2016). It is shorter for younger than for older listeners (Meister et al., 2018), and is increased for listeners with mild cognitive impairment (Lee & Lee, 2023). A decrease in VRT was shown by Gustafson et al. (2014) for noise reduction in hearing aids when turned on relative to off. Other findings are somewhat controversial. Pals et al. (2015) did not observe an effect of masker type, whereas Meister et al. (2018) found shorter VRTs for fluctuating than for stationary noise. Lewis et al. (2016) showed no effect of hearing impairment on VRT, but Meister et al. (2018) and McGarrigle et al. (2019) observed longer VRTs for hearing-impaired than for normal-hearing listeners. In addition, and in contrast to Gatehouse and Gordon (1990) and Cristiani and Alonso (2022), McGarrigle et al. (2019) found no effect of amplification. Based on these findings, it is expected that the VRT of young, normal-hearing listeners is shorter than that of older hearing-impaired listeners. However, the effects of masker type and amplification are unclear.

The relations between VRT and different measures of listening effort (physiological, behavioral, or self-reported) are weak or not significant (see e.g., Alhanbali et al., 2019; Mackersie & Cones, 2011; Strand et al., 2018). Meister et al. (2018) concluded that VRT was independent of SR-LE, whereas Visentin et al. (2022) found a correlation coefficient of 0.44 between the two measures.

In addition to VRT, also RSR can be assessed from response recordings. Schultheis and Jameson (2004) showed that compared to pupil size, the speech rate was better suited to estimate the cognitive load in a reading task. For more difficult texts, speech rate was lower than for easier texts, whereas pupil size showed no difference.

To the best of the authors’ knowledge, Lewis et al. (2016) is the only study that used the total duration of the response, i.e., VRT and the length of the utterance added together, as an additional measure to analyze listening effort for correct responses in a speech-recognition task. Groups of children with and without hearing impairment were compared at different SNRs and using different stimulus types (consonants, words, and sentences). The total duration of the responses decreased with increasing SNR. However, responses to consonants and words were not significantly different in total duration. Lewis et al. (2016) concluded that VRT is more useful than total duration to measure the effort for processing masked speech. Since the total duration in Lewis et al. (2016) included both VRT and the length of the utterance, the question arose whether the length of the utterance alone is suitable as a behavioral measure of listening effort under different listening conditions. However, since the length of the utterance depends on the number of repeated words, the speech rate was used in the current contribution instead of the length.

Whereas it is well known that speech rate depends on age, well-being, and physiological condition (e.g., Duchin & Mysak, 1987; Raming, 1983), changes in speech rate might depend on the conditions of the specific task. Kirchhübel et al. (2011) summarized studies on speech rate and stress that had produced inconsistent results. Most publications demonstrated an increase in speech rate with stress. However, there were exceptions, and prolongation of pauses as well as shortening of pauses were observed. When articulating speech during background noise, the findings are also ambiguous. For example, Charlip and Burk (1969) observed an increase in word duration with noise level, and Tuomainen et al. (2021) showed a decrease in articulation rate in background noises relative to the quiet condition, whereas Harmon et al. (2021) reported decreased pausing in different background noises. However, none of the studies evaluated RSR in adults of different ages during a speech-recognition task using different noise levels and different masker types. Based on the literature, it is hypothesized that RSR will increase with increasing SNR, will be higher for young normal-hearing listeners than for older listeners with hearing impairment, and might be dependent on masker type.

Previously, the measurement of response times like VRT required the design of complicated equipment. In the past ten years, VRT were measured using visual inspections of the recorded waveform (e.g., Gustafson et al., 2023; Lee & Lee, 2023; Lewis et al., 2016; McGarrigle et al., 2019; Pals et al., 2015; Visentin et al., 2022), by computer programs for response-onset detection (Cristiani & Alonso, 2022; Gustafson et al., 2014; Meister et al., 2018), or by ASR (Ibelings et al., 2022). Ooster et al. (2023) proposed a method for self-conducted speech audiometry using an ASR algorithm specialized in the analysis of responses of the German matrix test, i.e., the Oldenburg Sentence Test (OLSA; Wagener et al., 1999a, 1999b, 1999c). As a byproduct, the same ASR system enables an easy analysis of the listener's response times.

The current contribution focuses on the following hypotheses:

- ASR can be successfully applied to recordings of responses in a speech-recognition test. The ASR-based results for speech-recognition score and VRT are comparable to those of a human examiner, but allow easy extraction of additional information, such as RSR, for further analysis.
- Speech-recognition scores increase, and SR-LE is decreased, with increasing SNR. The degree of increase and decrease might depend on the masker type, but might not depend on the listener group.
- Speech-recognition scores are related to SR-LE. This relation might depend on the masker type and the listener group.
- VRT decreases and RSR increases with increasing SNR. Both measures depend on the listener group, and might show different results for stationary and for fluctuating maskers.
- VRT and RSR might be related to SR-LE effort.

## Methods

### Participants

Fifteen listeners with normal hearing (NH, eight males) and twelve listeners with sensorineural hearing impairment (HI, one male) with German as their first language participated in the study. The study was conducted as part of an unpublished thesis in which various maskers were generated and compared in terms of speech recognition and SR-LE of listeners. The size of the convenience sample was limited by the time available for data collection. The existing measurement data and recordings were re-analyzed as part of the current study.

**Figure 1.**
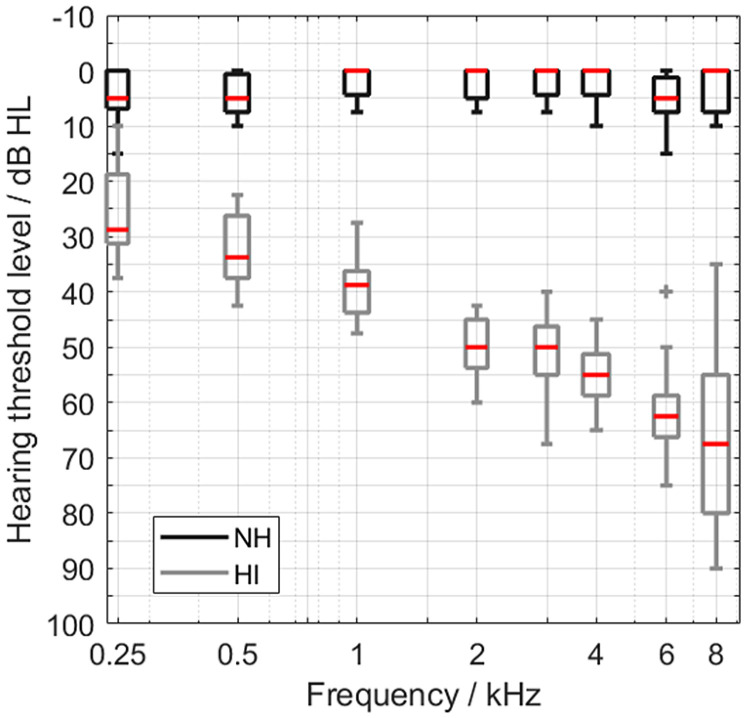
Pure-tone audiograms, averaged across left and right sides, for the two groups of 15 normal-hearing (NH, black) and 12 hearing-impaired listeners (HI, gray).

The NH group consisted of volunteer students with an average age of 25.5 (18–30) years. They had a hearing threshold level of 20 dB HL or less at each octave frequency in the range of 250–8 kHz and at 3 and 6 kHz. The HI group was composed of volunteers from the database of Hörzentrum Oldenburg, with an average age of 70.4 (43–81) years. Pure-tone audiograms of both groups measured with the audiometer DA930 (Kind, Großburgwedel, Germany) and headphones TDH39P (Telephonics, Farmingdale, NY, USA) are shown in Figure 1. All participants were invited for two sessions, received a compensation of 10 €/h, and gave their informed consent. The study was approved by the ethics committee (“Kommission für Forschungsfolgenabschätzung und Ethik”) of Carl von Ossietzky University Oldenburg, Germany.

### Equipment

Speech-recognition and listening-effort tests were performed with a PC running the Oldenburg Measurement Applications (HörTech gGmbH, Oldenburg, Germany) using an RME Hammerfall DSP multiface 24-bit sound card (Audio AG, Haimhausen, Germany), and HDA 200 headphones (Sennheiser electronic GmbH, Wedemark, Germany). For the NH group, a TDT HB7 headphone driver (Tucker Davis Technologies, Alachua, FL, USA) was used. This was replaced for the HI group by a Grapevine Head-Amp 4 (SPL Electronics GmbH, Niederkrüchten, Germany) that was able to provide higher output levels. The measurements were conducted diotically in a sound-insulated booth (Soundblocker, size b, Desone Modulare Akustik, Berlin, Germany). The participant's oral responses were recorded with a microphone e815 s (Sennheiser electronics GmbH, Wedemark, Germany) installed on a tripod inside the booth. The microphone was connected to channel 1 of an U46DJ 16-bit multi-channel sound card (ESI Audiotechnik GmbH, Leonberg, Germany), while the monitor output of the RME sound card was recorded on channel 2. Both signals were recorded using a second PC using the software Audacity.

### Stimuli

Speech recognition, SR-LE, and temporal structure of the responses were measured with the OLSA (Wagener et al., 1999a, 1999b, 1999c). The OLSA consists of five-word sentences with the fixed structure name-verb-number-adjective-object. This speech test is internationally known as a “matrix test,” as the ten different words available for each of the five words can be arranged in a matrix of 50 words in total.

As different maskers were compared in the original study, the measurement results were available for seven fluctuating and two stationary maskers:

- ICRA5-250: The masker ICRA5 (International Collegium of Rehabilitative Audiology, signal type 5; Dreschler et al., 2001) has the long-term average spectrum and temporal modulations of a single male speaker. The speech-pause durations in the ICRA5, which were longer than 250 ms, were shortened to 250 ms by Wagener et al. (2006).
- ISTS (International Speech Test Signal): The masker ISTS (Holube et al., 2010) is a mixture of recordings of six female speakers with characteristics of a single speaker and the long-term average spectrum of international female speech (ILTASS, Byrne et al., 1994). It has speech pauses with a duration of up to 650 ms. The ISTS has a length of 60 s, which is made up of sections with a length of 10 or 20 s.
- IFFM (International Female Fluctuating Masker): The sections of the ISTS were rearranged in their order and the speech-pause durations between 250 and 650 ms were shortened to 250 ms. After these modifications, the signal was again filtered to ILTASS.
- NFIMG (Native Female Informational Masker German): This masker contains the original recording of the German female speaker for the ISTS. Speech-pause durations longer than 250 ms were shortened to 250 ms. After this modification, the signal was filtered to ILTASS.
- NFIMM (Native Female Informational Masker Mandarin): This masker is the same as the NFIMG masker, but for the Mandarin female speaker of the ISTS.
- NFFMG (Native Female Fluctuating Masker German): This masker was generated with the same procedure as the ISTS from the original female recordings (see Holube et al., 2010), but only for the German speaker. The speech-pause durations were shortened to 250 ms.
- NFFMM (Native Female Fluctuating Masker Mandarin): This masker is the same as the masker NFFMG, but for the Mandarin female speaker of the ISTS.
- OLnoise (Wagener et al., 1999a, 1999b, 1999c): Stationary masker generated by multiple superpositions of the original recordings of the OLSA sentences spoken by a male speaker.
- IFnoise: Stationary masker generated by multiple superpositions of the ISTS.

The masker OLnoise is used as the standard with OLSA. To investigate the influence of the masking spectrum, the IFnoise was used as an alternative stationary masker, which, as a superposition of the ISTS, simulates the female spectrum of Byrne et al. (1994). Wagener et al. (2006) used the ICRA5-250 as a fluctuating masker in the OLSA. The main question of the original study was the use of the ISTS as a fluctuating masker and a comparison of the results with those with ICRA5-250 and the stationary maskers OLnoise and IFnoise. However, as ISTS has longer pauses than ICRA5-250, the pauses for the alternative masker IFFM were shortened to 250 ms. To avoid any confusion between the IFFM and the ISTS, the order of the sections with a length of 10 and 20 s that make up the ISTS was changed. If there are differences in speech recognition for ICRA5-250 and ISTS or IFFM, the question was whether these differences must be attributed to the different spectra or to informational masking. Hence, the intelligible masker NFIMG and the unintelligible masker NFIMM were used as further variants. These maskers have a comparable spectrum and a comparable pause length. In addition, the maskers NFFMG and NFFM were included to estimate the effect of the rearrangement of segments in the ISTS. The maskers ISTS, IFnoise, IFFM, NFIMG, and NFIMM are available from https://www.ehima.com/documents/. The maskers NFFMG and NFFMM are available on request from the authors.

All stimuli were free-field equalized. The maskers were normalized digitally to the same overall root-mean-square (RMS) level and for the NH group were presented at a long-term average level of 65 dB SPL.

For the measurement of listening effort for speech in the presence of the maskers, it was considered important that both the participants with and without hearing loss could perceive the maskers at a reasonable loudness. Hence, the sound-pressure level of the maskers was increased for the HI group by a gain in dB that was half of their individual hearing loss in dB at 500 Hz, but limited to a maximum presentation level of 80 dB SPL; it was reduced in 5-dB steps if the speech was perceived as too loud. This approach was chosen because, on the one hand, most maskers have a maximum in the frequency region of 500 Hz (Holube et al., 2010) and, on the other hand, the participants showed a high-frequency hearing loss. Since SNRs were chosen relative to the individual SRT (see description below) and therefore, speech recognition and listening effort of the participants with hearing loss did not have to be aligned as closely as possible to that of the participants without hearing loss at a fixed absolute SNR, frequency-dependent compensation of the hearing loss was not considered necessary.

### Speech Recognition Thresholds

The speech-recognition tests were performed using word scoring with test lists of OLSA consisting of 30 sentences each. The masker started 500 ms before each sentence, and ended 500 ms after each sentence. During the first session, all participants finished two training lists with the OLnoise masker at an SNR of 0 dB. The results were not analyzed. After the training, SRTs for a speech-recognition score of 50% were measured with the adaptive procedure of Brand and Kollmeier (2002) for all maskers and in randomized order. An open-test setting with word scoring was used, i.e., the participants repeated the perceived sentences orally without seeing the matrix of 50 words, and the experimenter confirmed every correct word on a PC screen.

### Speech-Recognition Scores and Self-Reported Listening Effort

The second session also started with two training lists of OLSA with the OLnoise masker and an SNR of 0 dB. For the following measurements, the speech material was divided into 45 blocks of five sentences each. Different blocks were presented at five different fixed SNRs. The SNRs were the individual SRT, and the individual SRT increased by 3, 6, 9, and 12 dB, respectively. For each participant, each of the nine maskers was used in combination with each of the five SNR levels, resulting in 45 sentence blocks (225 sentences). The presentation order of the sentence blocks was randomized, and different blocks were assigned to the respective conditions.

To allow analysis of the VRT, the maskers started 500 ms before each sentence, but ended 50 ms after each sentence. The earlier end of the maskers was intended to prevent some participants from waiting for the end of the maskers before starting their response, since that behavior would possibly level out potential differences between the measurement conditions in the VRT.

With the simple instruction “Bitte wiederholen Sie das Verstandene nach jedem einzelnen Satz” (engl.: “Please repeat what you have understood after each sentence”), the participants were asked to orally repeat the perceived speech. The participants were neither asked to respond as quickly as possible nor to ensure that their responses were as accurate as possible (Gustafson et al., 2023). The responses were immediately scored for speech recognition by the human examiner and additionally recorded for post-experiment analysis.

After each sentence block, the participants were asked to rate the perceived listening effort on a categorical scale (“Im Anschluss der fünf Sätze haben Sie die Möglichkeit, Ihre dabei persönlich empfundene Anstrengung zu kategorisieren”; engl. “At the end of the five sentences, you have the opportunity to categorize your personally perceived effort”). In contrast to Luts et al. (2010), the intermediate steps between their categories were not used. A numerical value was assigned to each category, but was not visible for the participant. Scale labels (and numbers in parentheses) were “mühelos” (1; engl.: no effort), “sehr wenig anstrengend” (2; engl.: very little effort), “wenig anstrengend” (3; engl.: little effort), “mittelgradig anstrengend” (4; engl., moderate effort), “deutlich anstrengend” (5; engl.: considerable effort), “sehr anstrengend” (6; engl.: very much effort), “extrem anstrengend” (7; engl.: extreme effort). The scale was presented to the participants on paper. The participants named the category of listening effort for each sentence block, and the experimenter noted the response. Since SR-LE was measured using a categorical scale, this variable was not considered suitable to analyze linear relationships (see statistical analysis). Hence, ranks were assigned to the assessments of SR-LE. The ranks were normalized using the proportion estimation formula Rankit (Soloman & Sawilowsky, 2009). Rank normalization was performed once for all listening-effort ratings (global), and once separately for the ratings of the individual participants. The aim of the latter method was to eliminate the different interpretation of the response scale by the participants, which can lead to a different distribution of responses, while maintaining the effect of SNR and masker type for each participant.

### Analysis with Automatic Speech Recognition

The recordings were analyzed offline after the experiment with the ASR system TDNN-LM5 proposed by Ooster et al. (2023). The system was implemented using the speech-recognition toolkit Kaldi (Povey et al., 2011). It used an acoustic model and a language model that had been trained especially for the OLSA speech material. In contrast to Ooster et al. (2023), the sentences presented were not used as additional knowledge of the ASR system during response analysis. Taking the presented sentences into account might have increased the likelihood of their detection by the ASR, but did not seem necessary. The output of the ASR was not a full transcription of the participant's responses, but rather the recognized words from the OLSA vocabulary (50 words in total), and one additional category “other words not in the OLSA vocabulary” (Out Of Vocabulary, <OOV>). This output was used to determine the speech-recognition scores of the ASR, which were compared to the manual examiner's scoring. For part of the statistical analysis (see below), ASR-derived speech-recognition scores were transformed to rationalized arcsine units (RAU scores; Studebaker, 1985).

In addition, based on the duration of its temporal windows, the ASR provided the temporal structure of the recordings, with a resolution of 30 ms. The results were independent of the analysis time of the ASR. The output information of the ASR contained the starting point and the duration of each word from the OLSA vocabulary, or was out of vocabulary.

### Verbal Response Time and Response Speech Rate

The VRT was defined as the duration between the end of a sentence and the start of the participant's response. In a manual approach, the VRT was first derived using an energy detector applied to the recordings. However, because of non-linguistic utterances of the participants (e.g., throat clearing) or other sounds (e.g., from movements of the participants), the results were not satisfying, and required subsequent manual corrections. In an alternative ASR approach, the starting point of the first word from the OLSA vocabulary or from OOV in the output of the ASR was used to measure VRT. These two approaches are compared in the results section.

In some recordings, the participants already responded before the end of the presentation. In the manual approach, the VRT was set to 0 ms for these recordings, while the ASR observed VRTs of up to −120 ms. This observation is in line with Pollack and Rubinstein (1963). To known words in background noise, they measured minimal reaction times of at least 300 ms after the onset of the word. Since mean word duration of the presentation in this experiment was approx. 450 ms, a minimum VRT of −150 ms was estimated. This minimum value was used as the reference when the VRTs were log-transformed with the natural logarithm to compensate for their skewed distribution (Whelan, 2008): 10·ln((VRT + 0.15 s)/0.15 s). Multiplication by 10 was used to transform the logarithmized VRT into a numerical range of 0–100. To eliminate the differences in response behavior between the participants, the log-transformed VRT was z-transformed within each participant, while maintaining the effect of SNR and masker type within each participant.

An additional variable was calculated from the ASR output, namely an estimate of the RSR of the participant's response. This was defined as the number of responded words divided by the duration from the starting point of the first word of the response to the end of the last word (from the OLSA vocabulary or OOV) in a sentence, given as words per second (wps). As with VRT, RSR was z-transformed within each participant to eliminate the differences in response behavior between the participants.

### Data Volume and Analysis

The presentation of five sentences at five SNR in nine maskers to 27 participants resulted in 6075 sentences. A few exceptions occurred during the analysis of these sentences: Three sentences were not recorded because the recording stopped after the response to the fourth sentence of the block. The speech-recognition scores of the ASR for these sentence blocks was increased by the average of the manual speech-recognition score for this block. As this correction was only applied to 0.05% of the sentences, its impact on the relationship between ASR and manual speech-recognition scores was considered to be negligible.

The participants gave no response to 22 sentences; hence manual and automatic VRT and RSR were not available for these sentences, while the recognition score was zero. For 52 sentences, the ASR detected at least one word, but the examiner classified the recording as “no response.” The reason for the difference may lie with the human examiner or with the incorrect analysis by the ASR. Thus, manual VRTs were not determined, while automatic VRTs and RSRs were available. For six sentences, the manual VRT was available, but not the automatic VRT. This was probably due to the low sound-pressure level of the responses. In summary, all data was available for 5992 of 6075 sentences. For all maskers and SNRs, the VRTs of at least one sentence were available. Hence, the analysis could be conducted without missing values.

### Statistical Analysis

Part of the results is given as boxplots that show the median as a horizontal line, a box from the 25th to the 75th percentile of the data, whiskers and outliers. All statistical analysis was carried out with IBM SPSS Statistics Version 28.0.1.1.

The SRTs for each masker and participant group from the first session were tested for normal distribution using the Shapiro-Wilk test. Differences in SRT between maskers were analyzed with an analysis of variance (ANOVA) and *t*-tests with repeated measures with the masker as within-participant factor.

The similarity between the manual and ASR speech-recognition score and between the manual VRT and ASR-derived VRT were analyzed with the intraclass correlation coefficient using a two-way mixed model of the type “absolute agreement.”

Possible differences between the participant groups were tested for significance for globally rank-normalized SR-LE, log-transformed VRT, and RSR using *t*-tests for independent samples.

The impact of SNR, masker type, and participant group on speech-recognition scores, SR-LE, VRT, and RSR were analyzed using linear regression models. For the variable masker type, the fluctuating maskers were assigned a 1 and the stationary maskers a 2. For the variable group, the participants with normal hearing were assigned a 1 and the participants with hearing impairment a 2. Prior to calculating the linear regression models, the assumptions linearity, no outliners, independence of the residuals, absence of multicollinearity, homoscedasticity of the residuals, and normal distribution of the residuals were tested. To achieve linearity, RAU scores were used for speech recognition. Since the Durbin-Watson criterion was violated for the globally rank-normalized SR-LE, the individually rank-normalized values were used for SR-LE. SR-LE is significantly correlated to SNR, violating the multicollinearity criterion. Therefore, these variables were not entered together in a linear regression model, but SNR was replaced by SR-LE in an additional linear regression model. Since the Durbin-Watson criterion was violated for the log-transformed VRT and RSR, linear regression models were used for the z-transformed logarithmized VRT and z-transformed RSR only. The independent variables were entered stepwise into the linear regression models. Linear regression models were preferred over Generalized Linear Mixed Models because the inclusion of participants as a random effect had little impact on the results.

The relations between the behavioral measures VRT and RSR and SR-LE were analyzed with Pearson correlation coefficients between the z-transformed and the rank-normalized measures at an individual participant's level.

## Results

### Speech-Recognition Thresholds

**Figure 2.**
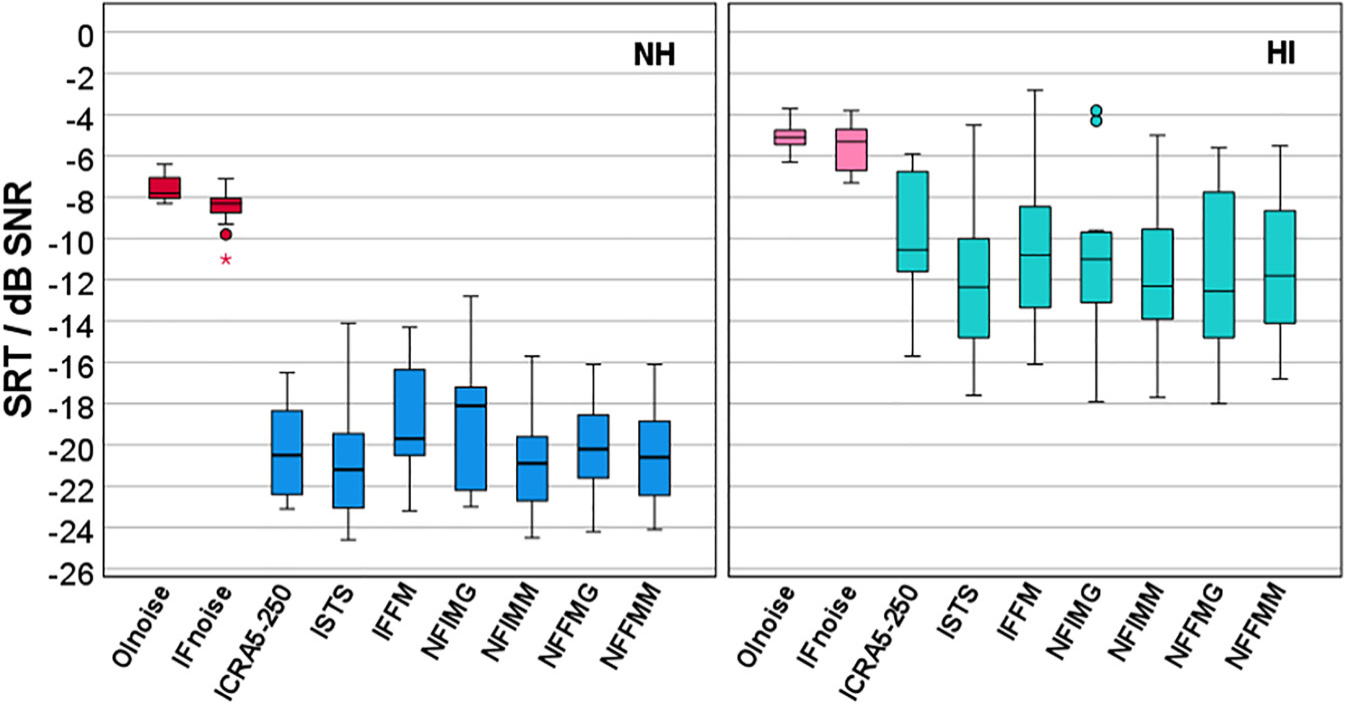
Boxplots of speech-recognition thresholds (SRT) for nine different maskers for participants with normal hearing (NH, left) and participants with hearing impairment (HI, right). Stationary maskers (Olnoise and IFnoise) are marked in red/pink and fluctuating maskers (ICRA5-250 to NFFMM, for abbreviations see methods section) are marked in blue/green.

Figure 2 shows boxplots of the SRTs for all maskers and both participant groups. The highest SRTs were observed for the two stationary maskers OLnoise (mean NH: −7.5 dB SNR, HI: −5.1 dB SNR) and IFnoise (mean NH: −8.5 dB SNR, HI: −5.5 dB SNR). Compared to the stationary maskers, the SRTs for the fluctuating maskers were reduced by about 12 and 6 dB for the NH and HI group, respectively.

Although the Shapiro–Wilk tests revealed that normal distribution was not fulfilled for masker NFIMG for the NH group (*p* = .044), but was for all other maskers and groups, parametric tests were applied. Significant differences between the participant groups and between stationary and fluctuating maskers were expected. Hence, the statistical analysis was limited to comparisons between maskers within each masker type, either stationary or fluctuating, and within each participant group, either NH or HI. The results of *t*-tests between the two stationary maskers OLnoise and IFnoise revealed a significant difference of 1 dB for the NH group (*t*[14] = 4.513, *p *< .001) but not for the HI group (*t*[11] = 1.512, *p *= .159). Mean SRTs for the fluctuating maskers in both participant groups were within a range of about 2 dB. For the NH group, the lowest SRT was observed for ISTS with −20.9 dB SNR and the highest SRT for IFFM with −18.8 dB SNR. An ANOVA showed a significant effect of the masker within the fluctuating maskers in the NH group (*F*[6, 84] = 3.482, *p *= .004). Pairwise posthoc *t*-tests with Bonferroni correction revealed only two significant differences, between ISTS and IFFM (*p *= .043) and between IFFM and NFIMM (*p *= .046). For the HI group, the lowest SRT was also observed for ISTS with −12.0 dB SNR and the highest SRT for ICRA5-250 with −10.1 dB SNR. An ANOVA showed a significant effect of the masker within the fluctuating maskers in the HI group (*F*[6, 66] = 2.944, *p *= .013). However, posthoc *t*-tests with Bonferroni correction were not significant. Because few significant differences in SRT were observed, and the respective differences were small, the measurement results of the two stationary maskers and the measurement results of the seven fluctuating maskers collected in the second session were combined in the further analysis, i.e., speech-recognition scores and rank-normalized SR-LE were averaged within the masker types, and for SR-LE without rank normalization, VRT, and RSR, the median was taken.

### Comparison Between Manual Analysis and ASR

**Figure 3.**
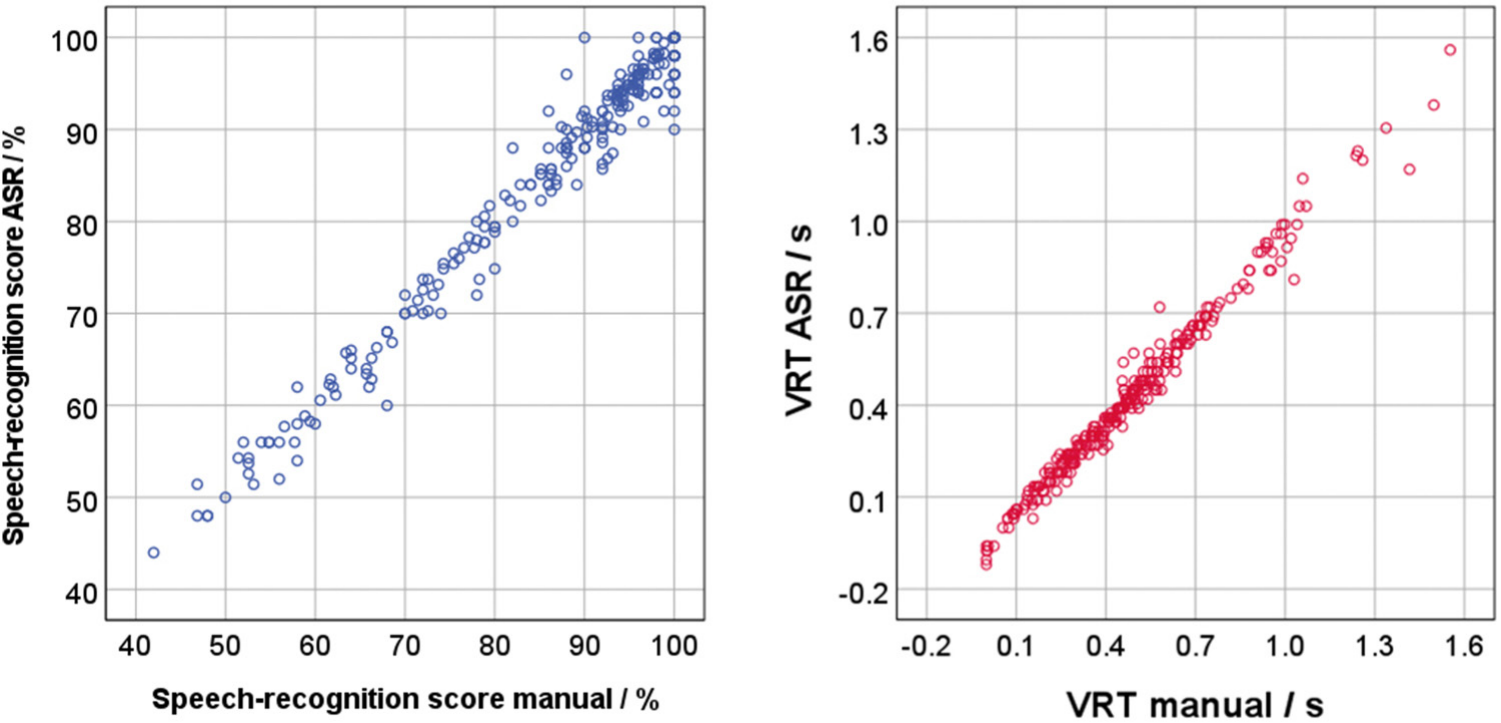
Comparison of speech-recognition scores (left) and VRT (right) of the human examiner (manual) and the automatic speech recognition (ASR). Each symbol represents the mean (speech-recognition score) or median (VRT) of 35 sentences in fluctuating maskers (a block of five sentences in each of the seven fluctuating maskers), or of 10 sentences in stationary maskers (a block of five sentences in each of the two stationary maskers) for one participant at one SNR (270 data points).

The comparison between the speech-recognition scores assessed from manual scoring by the examiner and by the ASR is shown on the left side of Figure 3. The right side shows the comparison between the manually- and ASR-measured VRT. Both comparisons demonstrated a satisfying match, with only few outliers. The intraclass correlation coefficient was 0.993 for speech-recognition scores, and 0.986 for VRT. For the following analyses, the speech-recognition scores and the VRT of the ASR were used.

### Speech Recognition and Listening Effort

**Figure 4.**
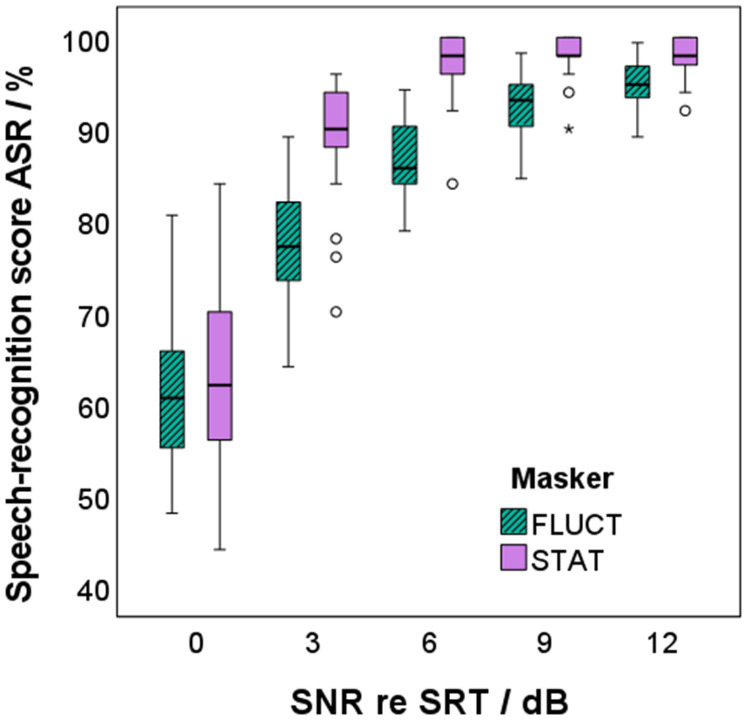
Speech-recognition score derived with the automatic speech recognizer (ASR), in relation to the signal-to-noise ratio (SNR) relative to the individual speech-recognition threshold (SRT) for stationary (STAT, purple) and fluctuating (FLUCT, green with diagonal shading) maskers.

Figure 4 shows the relation between speech-recognition scores and SNR relative to SRT for stationary and fluctuating maskers for all participants. Speech-recognition scores were RAU transformed for normalization of the distributions and a linear regression model was applied (see Model a) in Table 1). SNR and masker type, but not the participant group, were able to statistically significant predict RAU scores, *F*(3, 266) = 230.185, *p *< .001. Adjusted *R*^2^ increased from 0.615 for SNR as single factor to 0.719 when additionally including masker type into the regression model. The speech-recognition scores increased by 3.8 RAU with increasing SNR by 1 dB. This increase was stronger for stationary than for fluctuating maskers, i.e., a steeper slope of the psychometric function was observed for the stationary than for the fluctuating maskers. The speech-recognition scores of the stationary maskers were higher than for the fluctuating maskers by 13.4 RAU.

**Table 1.** Linear Regression Models for RAU-Transformed Speech-Recognition Scores (a and c), Individually Rank-Normalized SR-LE (b), Individually z-Transformed Logarithmized VRT (d), and Individually z-Transformed RSR (e).

	Unstandardized beta	Coefficients SE	Standardized beta	95% CI for beta		Significance of change
				Lower bound	Upper bound	
(a) RAU score						
SNR re SRT	3.813	0.157	0.785	3.504	4.123	<0.001
Masker type	13.390	1.333	0.325	10.765	16.015	<0.001
Group	−0.845	1.341	−0.020	−3.486	1.796	0.529
(b) Rank-normalized listening effort (individual)						
SNR re SRT	−0.174	0.006	−0.824	−0.185	−0.164	<0.001
Masker type	−0.663	0.047	−0.369	−0.756	−0.570	<0.001
Group	0.070	0.048	0.039	−0.023	0.164	0.140
(c) RAU score						
Rank-n. listening effort (individual)	−20.349	0.703	−0.887	−21.733	−18.964	<0.001
Masker type	−0.094	1.263	−0.002	−2.580	2.392	0.941
Group	0.587	1.182	0.014	−1.740	2.914	0.620
(d) z-transformed log(VRT)						
SNR re SRT	−0.183	0.006	−0.820	−0.195	−0.171	<0.001
Masker type	−0.679	0.052	−0.358	−0.781	−0.576	<0.001
Group	0.000	0.052	0.000	−0.103	0.103	1.000
(e) z-transformed RSR						
SNR re SRT	0.157	0.009	0.703	0.139	0.175	<0.001
Masker type	0.414	0.079	0.218	0.259	0.569	<0.001
Group	0.000	0.079	0.000	−0.156	0.156	1.000

**Figure 5.**
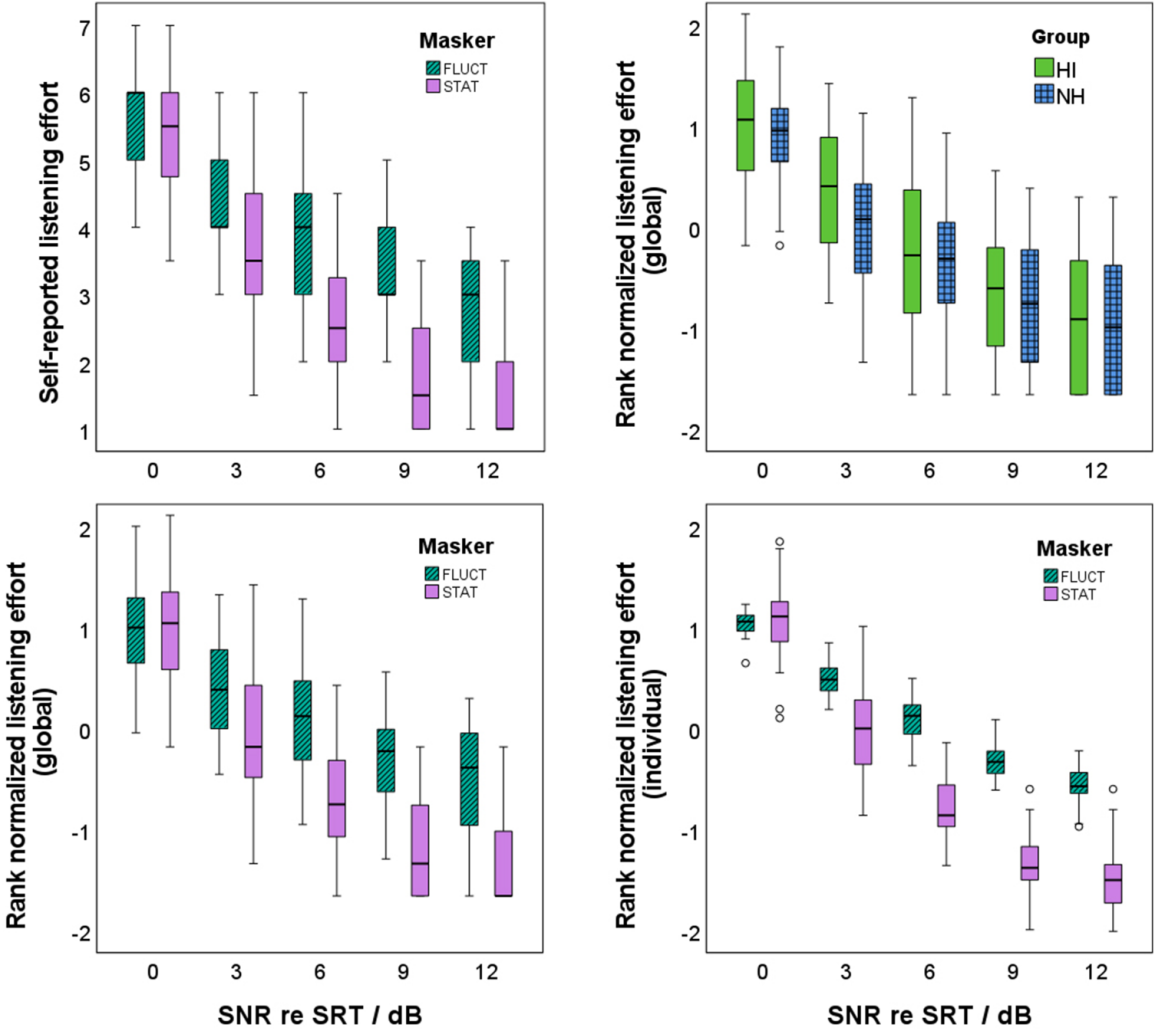
Self-reported listening effort (top left), rank-normalized self-reported listening effort based on all ratings for different masker types (bottom left) and different participant groups (top right), and based on individual ratings (bottom right) in relation to the signal-to-noise ratio (SNR) relative to the individual speech recognition threshold (SRT). Stationary masker (STAT) are given in purple, fluctuating maskers (FLUCT) are given in dark green with diagonal shading, young listeners without hearing impairment (NH) are given in light green, and older listeners with hearing impairment are given in blue with grid.

The relation between SR-LE and SNR relative to SRT is given in Figure 5. The distribution of the SR-LE on the categorical scale for stationary and fluctuating maskers (Figure 5, top left) is similar to the global rank-normalized SR-LE based on all ratings (Figure 5, bottom left). The comparison of the global rank-normalized SR-LE for the two participant groups (Figure 5, top right) revealed no significant difference (*t*[268] = −1.419, *p *= .157). If the rank normalization was performed at the individual level, the variance was reduced relative to the global rank normalization (Figure 5, bottom right). Table 1 gives the results for the linear regression model using the individually rank-normalized SR-LE (model b). SNR and masker type were able to statistically significant predict SR-LE, *F*(3, 266) = 393.724, *p *< .001. The participant group was already not a significant factor for the global rank-normalized SR-LE. This is all the more evident for the individual rank-normalized SR-LE. The adjusted *R*^2^ increased from 0.677 for SNR as single factor, to 0.813 when additionally including masker type into the regression model. Rank-normalized SR-LE decreased by 0.17 with increasing SNR by 1 dB. In analogy to the stronger increase in speech-recognition scores for stationary than for fluctuating maskers, this decrease in SR-LE was stronger for stationary than for fluctuating maskers. The rank-normalized SR-LE of the stationary maskers were lower than for the fluctuating maskers by 0.66.

**Figure 6.**
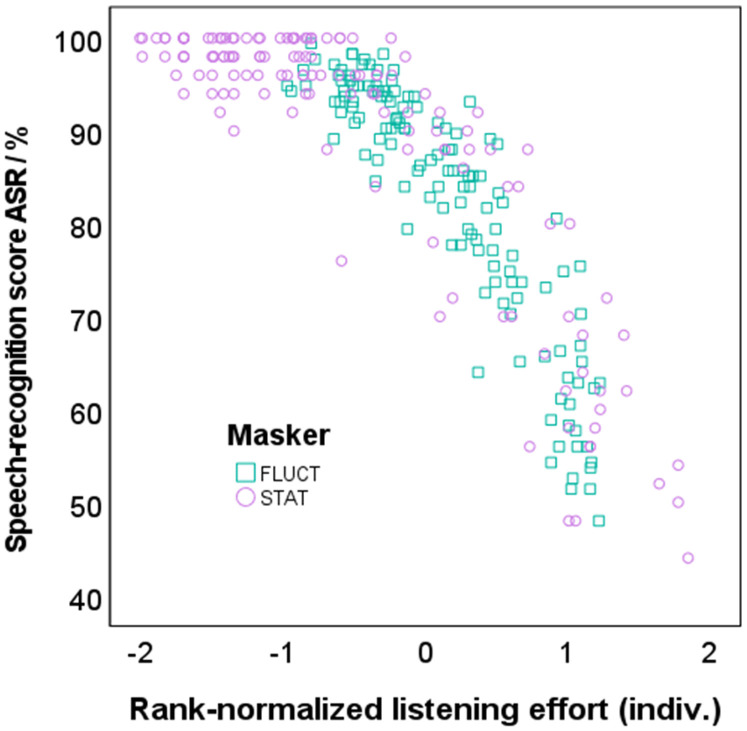
Relation between speech-recognition score derived with the automatic speech recognizer (ASR) and individually rank-normalized SR-LE. Results for stationary maskers (STAT) are given in purple circles and for fluctuating maskers (FLUCT) in green squares.

The relation between speech-recognition scores and SR-LE is given in Figure 6, and the corresponding regression model c) in Table 1. Rau-transformed speech-recognition scores were significantly related to individually rank-normalized SR-LE, while masker type and participant group did not contribute significantly in the model, *F*(3, 266) = 322.675, *p *< .001, adjusted *R*^2 ^= .782. SNR was not included into the model as it correlates strongly with SR-LE. The speech-recognition scores decreased by 20.3 RAU with increasing SR-LE by 1 on the individualized rank-normalized scale.

### VRT, RSR, and Self-Reported Listening Effort

**Figure 7.**
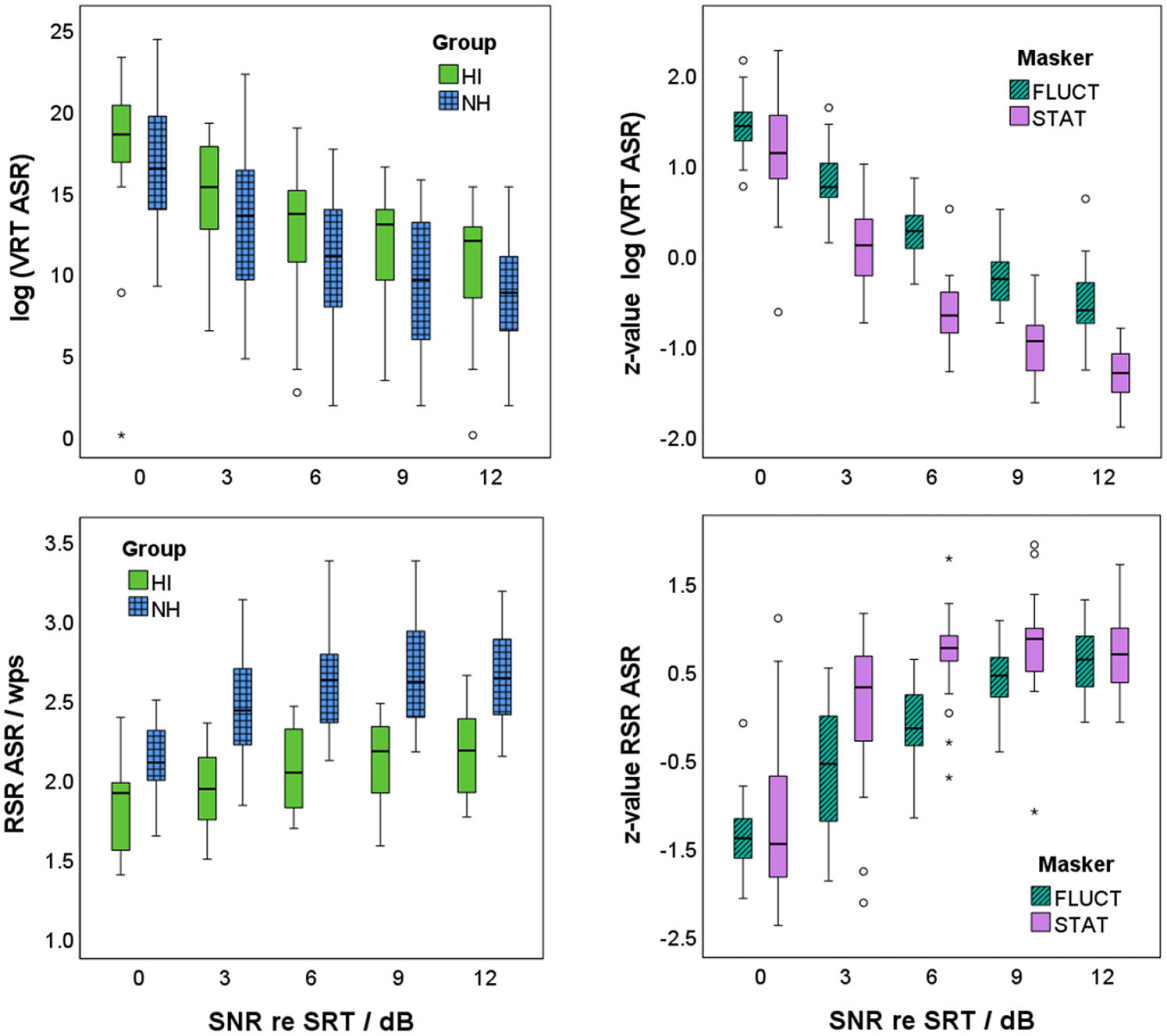
Verbal response time (VRT, top) and response speech rate (RSR, bottom) in dependence of the signal-to-noise ratio (SNR) relative to the individual speech-recognition threshold (SRT) for the two participant groups (participants with normal hearing: NH in blue with grid; participants with hearing impairment: HI in light green; left) and the two masker types (stationary maskers: STAT in purple; fluctuating maskers: FLUCT in dark green with diagonal shading; right). Note that log-transformed VRT and RSR are shown for the two participant groups, whereas *z*-values of the log-transformed VRT and of RSR are given for the two masker types. Seven outliners below zero in the upper left figure are not shown, to highlight the differences between the participant groups.

Log-transformed VRT decreased with increasing SNR relative to SRT as shown in Figure 7 (top left). Participants in group NH started their responses significantly faster than participants in group HI (*t*[268] = −2.990, *p *= .003). Log-transformed VRT were z-transformed within each participant, and a linear regression model was applied (see model (d) in Table 1). As shown in Figure 7 (top right), SNR and masker type, but not the participant group, were able to statistically significant predict VRT, *F*(3, 266) = 354.588, *p *< .001. Adjusted *R*^2^ increased from 0.671 for SNR as single factor to 0.798 when additionally including masker type into the regression model. The *z*-values of the log-transformed VRT decreased by 0.18 with increasing SNR by 1 dB. This decrease was stronger for stationary than for fluctuating maskers. The *z*-values of the log-transformed VRT of the stationary maskers were lower than for the fluctuating maskers by 0.68.

RSR increased with increasing SNR relative to SRT as shown in Figure 7 (bottom left). Participants in group NH articulated their response significantly faster than participants in group HI (*t*[268] = 12.170, *p *< .001). RSR were z-transformed within each participant, and a linear regression model was applied (see model e) in Table 1). As shown in Figure 7 (bottom right), SNR and masker type, but not the participant group, were able to statistically significant predict RSR, *F*(3, 266) = 104.744, *p *< .001). Adjusted *R*^2^ increased from 0.492 for SNR as single factor to 0.538 when additionally including masker type into the regression model. The *z*-value of RSR increased by 0.16 with increasing SNR by 1 dB. This increase was stronger for stationary than for fluctuating maskers. The *z*-values of the RSR of the stationary maskers were by 0.41 higher than for the fluctuating maskers.

**Figure 8.**
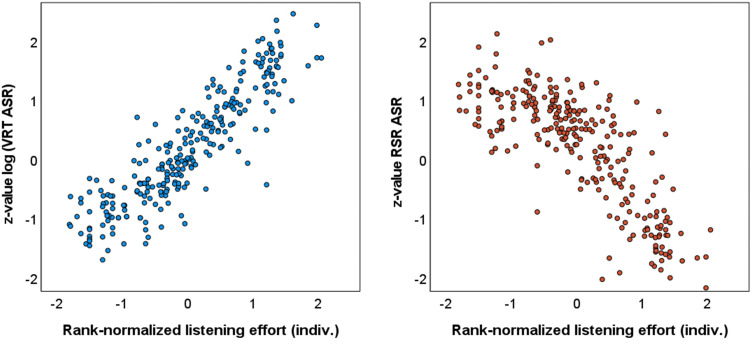
Relation between *z*-values of log-transformed verbal response time (VRT, left) and response speech rate (RSR, right) derived with the automatic speech recognizer (ASR) to individually rank-normalized SR-LE.

Figure 8 (left) shows a significant correlation between the *z*-values of the log-transformed VRT and the individually rank-normalized SR-LE. Correlation coefficients on an individual participant level were between *r *= .601 and *r *= .991 (mean *r *= .904). All correlations were significant with *p *≤ .005, except for one participant with *p *= .066. Compared to this, the correlation coefficients between the *z*-values of RSR and the individually rank-normalized SR-LE were lower. Correlation coefficients on an individual participant level were between *r *= −.382 and *r *= −.958 (mean *r *= −.788). Correlation coefficients were significant with *p *≤ .033, except for three participants with *p *= .130, .267, and .276.

## Discussion and Conclusion

In the experiment, young adults with normal hearing and older listeners with hearing impairment listened to matrix sentences in stationary and fluctuating maskers. In the first session, SRTs were measured with an adaptive procedure. In the second session, the stimuli were presented at five different SNRs. The SNRs were set to 0, 3, 6, 9, and 12 dB above the individual SRT, resulting in speech-recognition scores between 50 and 100%. In addition, SR-LE was rated on a categorical scale and the time course of the responses recorded. The SRTs of the two participant groups for stationary maskers were 2.4–3.0 dB apart, and both groups showed release from masking by dip-listening for fluctuating maskers. Furthermore, speech-recognition scores and SR-LE for both groups, NH and HI, were in the same range. It can therefore be assumed that the broadband increase in sound pressure level in group HI led to a comparable perception in both groups, i.e., no frequency-dependent amplification was necessary. As the differences within each of the two masker types for both participant groups were rarely significant and not practically relevant for this analysis, the results of the two stationary maskers and the seven fluctuating maskers were grouped. The results are discussed below in the light of the hypotheses.

### Comparison Between Manual Analysis and ASR

As expected, speech-recognition scores were mainly between 50% and 100%. However, speech-recognition scores in the area of 50% were rarely observed, although some of the measurements were performed at SRT. This is presumably the case, because the learning process in OLSA is not yet complete even after a training phase (Schlueter et al., 2016), and higher speech-recognition scores are achieved with repeated measurements.

Speech-recognition scores assessed using the ASR were highly correlated to the speech-recognition scores determined manually by the examiner. Complete agreement could not be expected, as errors can be made by both the ASR and the examiner. Nevertheless, the level of agreement is so high (intraclass correlation coefficient *r *= .993) that the ASR's speech recognition is deemed to be very satisfactory.

The same applies to the VRT. The only systematic deviation in VRT between ASR and examiner was observed for very low VRT values, because the examiner set all negative VRTs to zero, whereas the ASR also included negative VRTs. The shortest observed VRT of −120 ms is within the expected range of 150 ms shown by Pollack and Rubinstein (1963). Additionally, the value range of the VRT with an accumulation below 700 ms is also within the expected range. Hence, the ASR can be successfully applied to recordings of responses in a speech-recognition test. The ASR-based results for speech-recognition score and VRT are comparable to those of a human examiner, and compared to the manual analysis, the ASR greatly reduces the effort for the experimenter.

### Speech Recognition Scores and Self-Reported Listening Effort

The results showed that speech-recognition scores increased with increasing SNR. Difference in speech-recognition scores at SRT between stationary and fluctuating maskers due to dip listening (Moore, 2008) were not observed, as the measurements were performed at the individual SRT for the different maskers. However, the slope of the psychometric function was steeper for stationary than for fluctuating maskers, as also observed by Wagener and Brand (2005) and MacPherson and Akeroyd (2014).

Differences in speech-recognition scores between the groups NH and HI were also not expected for the measurements at the SNR corresponding to the individual SRT. However, differences may have occurred at SNR values above the SRT resulting from different slopes of the psychometric function. The analysis showed that speech-recognition scores did not differ between the two participant groups at higher SNRs either. This lack of significant effect may be attributed to the small participant groups. Nevertheless, the results did not reveal any effect of age or hearing impairment on the increase of speech-recognition scores with SNR relative to the individual SRT.

SR-LE was assessed on a categorical scale with seven labeled categories. Rank normalization of the ratings allowed them to be used as a normally distributed interval-scaled variable, and facilitated statistical analysis. If the ranks were assigned based on the ratings of all participants, then the distribution of the rank-normalized values were similar to those of the self-reported categories.

A comparison of the two participant groups based on the globally rank-normalized SR-LE revealed no statistically significant differences. This finding contradicts those of Larsby et al. (2005), Zekveld et al. (2011), and Krueger et al. (2017b). Based on their results, a higher SR-LE was expected for group HI than for group NH. Alternatively, based on Desjardins and Doherty (2013) and Zekveld et al. (2011), lower SR-LE could have been expected for group HI compared to group NH. The absence of a difference between participant groups may be attributed to stimulus presentations at SNRs relative to the individual SRT, i.e., higher SNRs for group HI than for group NH.

To eliminate the inter-individual differences between all participants in SR-LE, rank normalization was applied on an individual level. This reduction in variance can be attributed to the observation that the participants interpreted the categorical scale differently. They gave their ratings within different scale ranges, which increased the overall variance of the ratings, although each participant perceived a comparable relative change with SNR.

A linear regression model with individually rank-normalized SR-LE as the dependent variable revealed significant effects of SNR relative to SRT and masker type. The SR-LE decreased with increasing SNR. In agreement with Krueger et al. (2017b), the slope of the decrease was steeper for stationary than for fluctuating maskers. Hence, SR-LE is lower for stationary than for fluctuating maskers at the same relative SNR, supporting the findings of Hällgren et al. (2005), Larsby et al. (2005), Rudner et al. (2012), and Desjardins and Doherty (2013). The apparent contradiction with Krueger et al. (2017b), who found lower SR-LE and higher speech-recognition scores for fluctuating than for stationary maskers at the same SNR, is resolved when one considers that Krueger et al. (2017b) compared the ratings at the same absolute SNRs, whereas here the SNRs were selected relative to the individual masker-specific SRTs.

Transformation to individual rank-normalized values also reduced the variance when comparing speech-recognition scores to SR-LE. Both measures, speech-recognition scores and SR-LE, were significantly related, as also found by, e.g., Zekveld et al., 2010; Zekveld et al., 2011; Mackersie & Cones, 2011; and Krueger et al., 2017b. In contrast to the hypothesis, however, this relation was not dependent on the masker type, which was unexpected. However, since both the change in speech-recognition score and SR-LE with SNR depended on the masker type, the dependence on masker type was equalized when considering the direct correlation between speech-recognition score and SR-LE.

The significant correlation between speech-recognition scores and SR-LE raises the question of whether the participants rated listening effort, or replaced the instruction with a simpler one, such as performance (Feuerstein, 1992; Moore & Picou, 2018). A difference between SR-LE and performance was observed for SNRs below the SRT, where participants showed very low or no speech-recognition performance, but did not invest any effort as they give up (Pichora-Fuller et al., 2016). However, these conditions were not examined in the current study. Instead, a difference between SR-LE and performance, and thus an advantage of SR-LE, was observed at high SNRs: While speech-recognition scores, and hence performance, reached their maximum at 100%, SR-LE continued to decrease. However, this observation, which is in line with, e.g., Krueger et al. (2017b), does not prove that the participants were evaluating their SR-LE; they could also have been evaluating the difficulty of the listening situation (Rovetti, 2022).

### VRT, RSR, and Self-Reported Listening Effort

As expected from many previous studies, VRT decreased with increasing SNR and increasing speech-recognition score (Cristiani & Alonso, 2022; Gustafson et al., 2023; Ibelings et al., 2022; Lee & Lee, 2023; Lewis et al., 2016; McGarrigle et al., 2019; Meister et al., 2018; Pals et al., 2015; Visentin et al., 2022). This effect was observed even though the participants were not instructed to respond as quickly as possible (Gustafson et al., 2023). However, the trials did not follow a fixed schedule. After the participant’s verbal response, the examiner entered the words they had understood into the test interface. This was followed by the next presentation. The faster the participants responded, the earlier the test was completed. This timing may have motivated the participants to respond as quickly as possible.

Although the interindividual variance in VRT was very high, group HI showed significantly longer VRTs than group NH. The observation of longer VRTs for group HI is in keeping with Meister et al. (2018) and McGarrigle et al. (2019). However, it is in contrast to Gatehouse and Gordon (1990), who showed a shortening of VRT with hearing aid amplification. Although no hearing aids were used in the current study, the presentation level was increased for the group HI. Since Lewis et al. (2016) showed no effect between children with and without hearing loss, the differences between the two participant groups in the current study are probably due to age effects rather than an influence of hearing loss or presentation level.

VRT and SR-LE therefore differ in terms of the age effect. While the participant group is a significant factor for VRT, this is not the case for SR-LE. This difference could complicate future studies if VRT is used with participants of different age and hearing abilities. However, when VRT is used as a relative measure within participants and differences between participants are eliminated, for example, by z-transformation, VRT seems to be suitable for comparing different conditions.

The linear regression model for z-transformed VRT showed significant effects of SNR relative to SRT and masker type, but not for participant group. The latter result was expected, as the z-transformation was performed within participants to eliminate individual differences in reaction times. VRTs were significantly longer for the fluctuating maskers than for the stationary maskers for the same SNR relative to SRT. This finding was in line with the results for speech-recognition scores and SR-LE, i.e., higher speech-recognition scores and lower SR-LE for stationary than for fluctuating maskers. However, it contradicts the results of Pals et al. (2015) and Meister et al. (2018). Pals et al. (2015) did not show a significant difference in VRT between a stationary and a fluctuating masker (8-talker babble). It is noteworthy that they measured at two SNRs: The SRT for a speech-recognition score of 79% (instead of 50% in the current study) and an SNR increased by 5 dB relative to that SRT. Since the SRT was determined for each masker separately, the measurements at the SRT for 79% were performed at the same speech-recognition score. A similar approach was followed by Meister et al. (2018), who showed shorter VRTs for a fluctuating, relative to a stationary, masker. They compared the VRTs for the two maskers at speech-recognition scores of 80% and 95%, which were determined for both maskers separately. Hence, if we had compared the VRTs at the same speech-recognition score and not at the same SNR relative to SRT, the VRTs for the stationary and fluctuating maskers may have been aligned, or the VRT for the fluctuating maskers may even have been lower than those for the stationary maskers. This assumption is supported by comparable VRTs for both masker types at SRT, and similar differences at higher SNRs.

The results for RSR showed a similar (but opposite) trend as for VRT: RSR increased with increasing SNR relative to SRT. Participants in group HI articulated their response significantly slower than those in group NH. The decrease of RSR with age is well known (e.g., Duchin & Mysak, 1987; Raming, 1983) and has been attributed in the literature to a general neuromuscular slowdown. It is unclear, however, how much hearing impairment contributes to the effect.

The linear regression model for z-transformed RSR showed significant effects of SNR relative to SRT and masker type, but not for participant group. The latter result was again expected, as the z-transformation was performed within participants. RSRs were larger for stationary maskers than for fluctuating maskers. The effect of masker type was observed in the current contribution for the first time, and therefore cannot be compared with the literature. The decrease in RSR with decreasing SNR, i.e., increase in task difficulty, is consistent with Schultheis and Jameson (2004), Lewis et al. (2016), and several other studies outside hearing research (see, e.g., Kirchhübel et al., 2011). However, it is noteworthy that RSR as a measure of articulation speed did not increase over the entire SNR measurement range. Rather, at higher SNRs, RSRs did not increase any further, i.e., the participants seemed to have reached their fluent articulation rate. Due to this apparent limitation of RSR, and the higher explained variance in the regression model for VRT (*R*^2 ^= .798 for VRT compared to *R*^2 ^= .538 for RSR), it can be concluded in accordance with Lewis et al. (2016), that as a behavioral measure in a single-task speech test, VRT is better suited than RSR.

In the current contribution, VRT and RSR were both significantly correlated to SR-LE. The absolute values of the correlation coefficients were on average higher for VRT than for RSR (|*r*| = .904 for VRT and |*r*| = .788 for RSR). This finding supports the previous conclusion that as a proxy of SR-LE, VRT is better suited than RSR. The significant linear relation between VRT and SR-LE is unexpected, because relations between different measures of listening effort are typically weak or absent (e.g., Alhanbali et al., 2019; Mackersie & Cones, 2011; Meister et al., 2018; Strand et al., 2018; Visentin et al., 2022). It should be noted that individually z-transformed VRTs, and individually rank-normalized SR-LE, were used to analyze the correlation. The transformations, which were possible due to repeated measurements under different conditions, eliminated interindividual differences in self-reports and in reaction times, and hence only relative changes across the conditions were considered. However, other measures of listening effort, such as pupillometry, have similar limitations, namely the observation of inter-individual differences that are compensated for by baseline determination.

The linear relationship between VRT and SR-LE indicates that the two variables can be used interchangeably. Since the VRT can be determined automatically from the participant's responses, additional assessments of the subjectively perceived listening effort are unnecessary. However, both variables increase with SNR, i.e., with the difficulty of the listening situation. This raises the question as to what additional information VRT or SR-LE provide. The advantages of VRT or SR-LE over SNR are revealed in the analysis of the effects of different masker types as in the current study, or will possibly be revealed in future studies on the benefits of technical hearing devices or other interventions. So far, the benefit is typically quantified by a change in SRT, which can be determined very precisely using adaptive methods. In an alternative approach, measurements could be carried out at higher and more realistic SNRs, where speech-recognition scores are close to or in saturation. In such listening conditions, the VRT or the LE could resolve differences between interventions.

The results allow the following summary and conclusion: not only is a specially trained ASR system suitable for the automated assessment of the correctness of participants’ responses, but it is also possible to derive a proxy for SR-LE directly from the temporal response behavior of participants during speech audiometry. To reduce inter-individual differences in SR-LE and VRT, it is recommended to standardize these variables individually using a z-transformation or rank normalization.
